# Management of Liver Hydatid Cysts: A Retrospective Analysis of 293 Surgical Cases from Southern Iran

**DOI:** 10.1155/2023/9998739

**Published:** 2023-06-19

**Authors:** Amirhossein Erfani, Reza Shahriarirad, Mehrdad Eskandarisani, Mohammad Rastegarian, Bahador Sarkari

**Affiliations:** ^1^Thoracic and Vascular Surgery Research Center, Shiraz University of Medical Sciences, Shiraz, Iran; ^2^Student Research Committee, Shiraz University of Medical Sciences, Shiraz, Iran; ^3^Basic Sciences in Infectious Diseases Research Center, Shiraz University of Medical Science, Shiraz, Iran; ^4^Department of Parasitology and Mycology, School of Medicine, Shiraz University of Medical Sciences, Shiraz, Iran

## Abstract

**Background:**

The current study aimed to evaluate the therapeutic features and complications of liver hydatid cyst in patients who underwent surgery for cystic echinococcosis (CE) in Fars province, southern Iran.

**Methods:**

A total of 293 patients who underwent surgery for liver hydatid cyst from 2004 to 2018 in Fars province, southern Iran, were retrospectively evaluated. The clinical records of patients were reviewed, and the demographic and clinical characteristics of each patient were assessed.

**Results:**

Of the total of 293 cases, 178 (60.9%) were females and 115 (39.1%) were males. The mean age of the subjects was 37.22 (±20.55) years. The mean size of the liver hydatid cyst was 9.18 (±4.365) cm. Of the 293 patients studied, 227 (77.4%) had hydatid cysts only in the liver, while 55 (9.4%) had both liver and lung cysts. More than half of the liver cysts (65.9%) were located in the right portion of the liver (segment 5 to 8). Of the 293 cases, 52 (17.7%) underwent radical surgery, while 241 (82.3%) underwent conservative surgery. Recurrence of hydatid cyst was recorded in 46 (15%) of cases. Patients who were treated with radical surgery in comparison with those who had conservative surgery had a lower recurrence rate but a longer duration of hospital stay (*P* < 0.05).

**Conclusion:**

Recurrence remains as one of the major challenges in the management of hydatid cyst. Radical surgery reduces the chance of recurrence, although this procedure increases the length of hospital stay.

## 1. Introduction

Cystic echinococcosis (CE) is a worldwide zoonotic parasitic disease caused by the larval stage of *Echinococcus granulosus.* The disease is considered to be a burden on public health in many countries, especially in temperate zones such as central Asia, the Middle East, Australia, and South America [1]. CE can affect almost all organs in the body but usually affects the liver (50–70%) followed by the lungs [2–4]. Signs and symptoms of the liver hydatid cyst vary and depend on the location and size of the cystic structure. The cyst may be asymptomatic at the early stages of infection and become symptomatic if the cystic structure grows larger or becomes complicated. In symptomatic cases, it may present with abdominal pain, mostly in the right upper quadrant, jaundice, nausea, vomiting, and abnormal liver function tests.

There are several treatment options for human CE including surgical and nonsurgical management, depending on the size, location, and existence of complications in the cystic structure [5, 6]. Till about four decades ago, surgery was the only treatment for human CE; however, in the past few years with the advent of new techniques, several other treatment options have been introduced such as chemotherapy with benzimidazole and PAIR (percutaneous aspiration and injections of protoscolocidal chemicals and respiration) procedure [7–10]. However, the removal of an intact hydatid cyst, if possible, remains as one of the best treatment options since it usually leads to immediate and complete cure with little chance of recurrence [11]. Radical treatment which is removing the whole cyst “en bloc” is considered to be the appropriate procedure for the management of the liver hydatid cyst, yet simple drainage, marsupialization, capitonnage, and resection of a part of the involved organ may be used depending on the condition, size, location, and existence of complications [11].

The current study aimed to evaluate the surgical treatment and complications of the liver hydatid cyst in patients who underwent surgery in two main hospitals of Shiraz, in Fars province, southern Iran, where CE is a real health challenge [12].

## 2. Methods and Materials

### 2.1. Study Area

Fars province with the capital of Shiraz in one of the thirty-one provinces of Iran located in southwest part of the country with the population of more than 4.6 million people, of which 67.6% live in urban areas, 32.2% in rural areas, and 0.3% live as nomad tribes. The average temperature of Fars province is 17.8°C, ranging between 4.7°C and 29.2°C, and the annual rainfall is 261 mm. Animal farming and agriculture are of great importance as they are considered as two main economic bases of this province. This type of livelihood has facilitated the presence of some of zoonotic diseases such as CE, toxocariasis, and leishmaniasis [13–16].

### 2.2. Ethics Approval and Consent for Publication

The study was approved by the Ethical Committee of Shiraz University of Medical Sciences (SUMS) and has been performed in accordance with the ethical standards version of the Declaration of Helsinki. The patients' records were anonymized and deidentified prior to analysis. The confidentiality of the details of the subjects was assured.

### 2.3. Data Collection

The study has been reported in line with the STROCSS criteria [17]. Hospital records of a total of 293 patients who underwent surgery for the liver hydatid cyst from 2004 to 2018 in two major university-affiliated hospitals in Fars province, southern Iran, were retrospectively evaluated. In all cases, the preliminary diagnosis of hydatid cyst has been confirmed by postoperative pathological findings. So, the inclusion criterion of operation with the diagnosis of CE was considered while reviewing the records.

Information such as age, sex, presenting signs, and symptoms of hydatid cyst; number and the specific location of cystic structures; history of relapse; reoperation; characteristics of the cyst (calcification, puss discharge, multicystic, multiloculated, daughter cyst, rupture, inflammation, superimposed infection, and septation); early postoperative complications; and diagnostic procedures such as ultrasound scanning, computed tomography, X-rays, pathology reports, lab data, operation information, and drug therapy were extracted from the hospital records of each patient. The surgical procedure used for the patients, radical or conservative, was noted. Radical surgery is mainly defined by resecting a part or entire organ to remove the cystic structure. Subadventitial cystectomy is also considered as a radical procedure. Conservative surgery is considered as a simple, easy-to-perform procedure that aims to remove the parasitic content of the cyst, without completely removing the cystic (fibrotic) wall; such procedure is also known as a deroofing or resection of the protruding dome, sometimes associated with omentoplasty.

### 2.4. Statistical Analysis

All data were recorded, using SPSS software, version 22 for Windows (SPSS Inc.). Statistical analysis was performed using the chi-square test for independence or categorical dependent variables. *P* < 0.05 results were considered to be statistically significant.

## 3. Results

The total number of patients who underwent surgery with hydatid cyst between 2004 and 2018 was 293 cases. Of these, 178 (60.9%) were females and 115 (39.1%) were males. The mean age of the subjects was 37.22 (±20.55) years, ranging from 3 to 96 years. Most cases (31.4%) were in the age group of 50 years and older. Of 293 patients, 126 (43%) presented with abdominal pain, 26 (8.9%) with nausea, 21 (7.2%) with vomiting, 8 (2.7%) with weight loss, 23 (10%) with anorexia, 35 (15.4%) with fever, 9 (4%) with chills, 16 (7%) with dyspnea, and 20 (8.9%) with cough. On examination, 9 (4%) had tenderness, 11 (4.8%) reported mass sensation on palpation, and 5 (1.7%) had organomegaly. The median duration of symptoms among our patients was 90 [Q1–Q3: 30–232] days, in which 20 (17.2%) had up to one week of symptoms, 18 (15.5%) between one week and one month, 26 (22.4%) between one month and 90 days, and 52 (44.8%) above 90 days.

**Figure 1:**
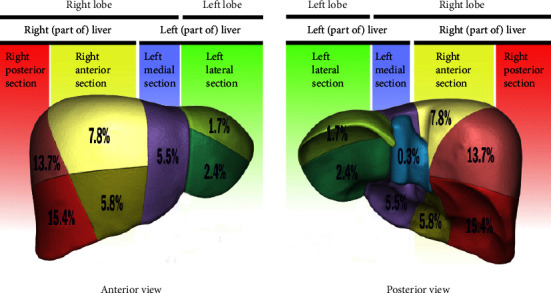
Rate of hydatid cyst infection (percent) in different segments of the liver.

Imaging results showed that the liver hydatid cyst was detected in 138 out of 139 (99.3%) by abdominal CT scans and also detected in 187 among 189 (98.9%) by abdominal ultrasound imaging. Among the 293 cases, 170 (67.5%) cases had a single cyst, 52 (20.6%) had 2 cysts, and 30 (11.9%) had 3 or more cysts. Of the 293 patients studied, 228 (77.8%) had hydatid cysts only in the liver, while 51 (17.4%) had both liver and lung cysts. We also observed 19 (6.5%) cases of hydatid cysts of the liver along with other locations including two cases in the lung and spleen, one case in the lung and pelvic cyst, one case in the lung and subdiaphragmatic area, one case in the lung and pancreas, one case in the spleen and pelvic cavity, three cases in the spleen, three cases in the pelvic cavity, two cases in the subdiaphragmatic area, two cases in the abdominal and peritoneal cavity, one case in the psoas muscle, one case in the mediastinum, and one case in the kidney. It is worth mentioning that there was a significant association between having the hydatid cyst in the liver and lung (*P* < 0.05) but not between the liver and other locations (*P* > 0.05). More than half of the liver cysts (65.9%) were located in the right portion of the liver (segment 5 to 8), while 82 (28%) of them were located in the left lobe (segment 1 to 4).  Figure 1 shows the percentage of infection with hydatid cyst in various liver segments, and Table 1 shows the frequency and correlation of the hydatid cyst in different segments of the liver lobes.

The size of the liver hydatid cyst ranged from 1 to 26 cm (mean: 9.18 and SD = 4.365). Most of the cysts (50.6%) were between 6 and 10 cm, and the least cases [10] were above 15 cm. The area of square meters of the liver cysts ranged from 1 to 393 cm^2^ (mean = 60.41 and SD = 54.259) with the highest frequency (31%) in less than 26 cm^2^ and the least (13.5%) in the 51 to 75 cm^2^ group.

Of the 293 cases, 52 (17.7%) underwent radical surgery, while 241 (82.3%) underwent conservative surgery. Patients who were treated with the radical surgery method had a lower recurrence rate (23.7% versus 76.3% for conservative surgery) but longer duration of hospital stay (8.14 versus 10.82 days). There was a statistically significant association between the type of surgery and the length of hospital stay (*P* < 0.05). Also, the median duration of hospital stay for patients was 6 [Q1–Q3: 4–10] days with the highest frequency in less than 5 days (127 cases; 45.2%) and the least frequency for 31 days and above (5 cases; 1.8%).

Of the 293 liver hydatid cysts, 35 (11.9%) were calcified, 27 (9.2%) had daughter cysts, 9 (3.1%) were ruptured, 3 (1%) had puss discharge, 3 (1%) were multicystic, and 21 (7.2%) were multiloculated. Also, 4 (1.4%) had fibrosis, 4 (1.4%) had inflammation, 14 (4.8%) had superimposed infection, and 9 (3.1%) had septation.

In terms of medical laboratory data, 51.9% of patients had leukocytosis on admission in which 63.3% had neutrophilia, 1.7% had lymphocytosis, 62.5% with eosinophilia, and 11.8% had monocytosis. Furthermore, a summary of laboratory data can be seen in Table 2.

Regarding the drug treatments, various combined medications were used during hospitalization. Anthelmintic drugs (albendazole) have been used in 55.3% of cases. Other medications included third-generation cephalosporins (64.5%), fluoroquinolones (24.6%), first-generation cephalosporin (24.6%), lincosamide (16.7%), aminoglycosides (3.8%), glycopeptides (1.7%), *β*-lactam (1.7%), carbapenem (1.7%), second generation cephalosporin (0.3%), and sulfonamides (0.3%).

Recurrence of the hydatid cyst was recorded in 46 (15%) of cases, of which 32 (10%) occurred once, twice in 4 (0.01%) cases, and three times in 2 (0.006%) cases. In patients with recurrent disease, 23.7% had undergone radical surgery, while 76.3% had conservative surgery treatment. It should be noted that based on the information which was available in the patients' records, it was not clear whether the recurrence of the disease was due to the cavity-related complications or the reactivity of the parasite. Accordingly, the actual recurrence rate associated with parasite reactivation may be less than the rate stated here.

## 4. Discussion

Hydatid cysts remain a major health challenge in many countries of the world, including Iran [12, 18–20]. In this study, the clinicohematological and therapeutic features of hydatid cyst cases admitted to university-affiliated hospitals, from 2004 to 2018, in Fars province, southern Iran, were reviewed.

Based on our findings, abdominal pain followed by fever, anorexia, nausea, and vomiting were the most common symptoms of patients before admission. Other studies reported almost the same results with abdominal pain being the most common symptom of the liver hydatid cyst [21–23]. Motie et al. reported that patients with the liver hydatid cyst presented with abdominal pain followed by fever in most cases [24]. In their study, organomegaly and palpable mass were considered as the most common finding in physical examination, while in our study, the most common finding in the examination was abdominal tenderness [24].

As liver CE is a chronic disease and symptoms would show up when the cystic structure becomes large enough to be complicated or pushes the adjacent organs, patients usually do not develop the acute symptoms. In this study, most of the patients had symptoms more than 90 days before the admission which is similar to the findings of Tagliacozzo et al., a study in Italy which reported duration of symptoms of 2 up to 72 months (mean 16 months) for patients with liver CE before the admission to the hospital [21].

The establishment of the correct diagnosis of hepatic hydatid cyst is essential before the operation and is based upon clinical, radiological, and laboratory data. Differential diagnosis relies greatly on imaging techniques such as ultrasonography, X-rays, and computed tomography scans. Ultrasonography is considered to be the gold standard method for diagnosis of the disease, while the CT scan is often used in emergency presentations [4]. In a study by Prousalidis and colleagues, the liver hydatid cyst was found in 92% and 95% of the ultrasonographies and CT scans which is in line with our findings [25]. In Prousalidis et al. study, leukocytosis was found in all cases and hypereosinophilia in 14.3% of them, while in our study, almost half of the patients had leukocytosis and more than half of them had hypereosinophilia [25].

New procedures have been used in recent years for the management of hydatid cyst including total cystectomy and percutaneous and perendoscopic procedures, which improved the treatment efficacy as well as the quality of life of CE patients [22]. Management of liver hydatid cysts includes percutaneous sterilization practices, surgery, drug treatment, a “watch-and-wait” approach, or a combination of these [24]. In our study, most of the cases (82.3%) underwent conservative surgery. Patients who were treated with the radical surgery method had a lower recurrence rate but longer duration of hospital stay. Motie et al. and Gomez et al. recommended the radical surgery treatment because of the shorter length of hospital stay and its lower rate of complications and recurrence [24, 26]. Tagliacozzo and collogues reported that although radical surgery can reduce the rate of complications, recurrence, and hospital stay, performing a radical surgery must be done only in an exceptional case to avoid removing healthy hepatic parenchyma [21].

The effectiveness of radical treatment in comparison with conservative treatment has been documented in several studies. In Georiou et al. study, the therapeutic features of 232 patients who underwent surgery for liver hydatid disease were evaluated. Those patients who underwent a radical procedure had no mortality and no recurrence and a low rate (10.95%) of morbidity. Those patients who were treated with the conservative method had a mortality rate of 2.76% and morbidity of 24.13% and 6.9% of relapse at three-year complete follow-up. The authors concluded that radical surgical treatment are better tolerated by patients and yielded better results in terms of mortality and the rate of recurrence [27]. In Secchi et al. study in Argentina, a relatively large group of CE patients (1412) with radical or conservative surgical procedures has been evaluated. The complication rate has been significantly lower in patients with radical surgery compared with the other procedures. Moreover, the rate of reoperation and recurrence has been significantly lower in patients with radical surgery treatment [23].

Biliary spillage is the foremost common cause of postoperative morbidity after conservative liver CE surgery. In Surmelioglu et al. study, postoperative leakage was detected in 36 out of 186 (19.4%) patients with solitary liver hydatid cyst with conservative surgery [26]. While conservative surgery with omentoplasty is reducing the postoperative complications, radical surgery along with administration of albendazole has been considered as the best management option for the liver hydatid cysts owing to its low number of complications and recurrence [28].

Recurrence remains one of the main complications in the management of hydatid disease. In the current study, recurrence of hydatid cyst was recorded in 15% of cases, of which 10% occurred once and 0.01% and 0.006% cases occurred twice and three times, respectively. In the recurrence cases, 23.7% had been treated with radical surgery, while 76.3% with conservative surgery. In another study by the authors in Yasuj district in southwest of Iran, recurrence has been reported in 14% of hydatid cysts [20]. In Spain, among the 217 patients with the liver hydatid cyst, 25 (11.5%) had a hydatid recurrence after curative treatment [29].

Anthelmintic drugs (mostly albendazole) are the drug of choice which are often used in liver CE, and it appears to have greater efficacy (20–60%) for shrinkage or disappearing the cystic structure than any other agents used so far [18, 30]. In a systematic review and meta-analysis, Gomez et al. reported that anthelmintic drugs alone are not considered as ideal treatment for hydatid cyst of the liver [26]. In our study, the main drugs that have been used were albendazole and third-generation cephalosporins. The use of the third-generation cephalosporin (especially ceftriaxone) is because of their prophylactic effects, after the surgery, for the prevention of wound infection and complications.

Given the nature of any retrospective study, our study also had shortcomings. These limitations included some missing data and the inability to follow up the patients. Also, we only included operated cases of hydatid cyst, and therefore, our study population is not representative of the total case in our province including those who were managed medically or through noninvasive procedures such as puncture aspiration injection reaspiration (PAIR). Furthermore, postdischarge mortality and complications were not evaluated in our study due to the retrospective nature of our report.

## 5. Conclusion

Hydatid cyst, especially liver disease, as the most common form of the disease, is still a significant health problem in Iran. Despite advances in the treatment of the liver hydatid cyst, there are several challenges that exist including choosing the appropriate surgical procedure based on the individual patient's condition. Recurrence remains as one of the major challenges in the management of this disease. Radical surgery reduces the chance of recurrence, yet increasing the length of hospital stay.

## Figures and Tables

**Table 1:** The frequency and correlation of the hydatid cyst in different segments of the liver lobes.

	Seg. 1	Seg. 2	Seg. 3	Seg. 4	Seg. 5	Seg. 6	Seg. 7	Seg. 8
Seg. 1	1 (0.3%)	0 (0%)	0 (0%)	0 (0%)	0 (0%)	0 (0%)	0 (0%)	0 (0%)
Seg. 2	0 (0%)	5 (1.7%)	2 (0.7%, *P*=0.005)	0 (0%)	0 (0%)	1 (0.3%, *P*=0.568)	0 (0%)	0 (0%)
Seg. 3	0 (0%)	2 (0.7%, *P*=0.005)	7 (2.4%)	1 (0.3%, *P*=0.328)	1 (0.3%, *P*=0.345)	1 (0.3%, *P*=1.00)	1 (0.3% *P*=1.00)	1 (0.3%, *P*=0.439)
Seg. 4	0 (0%)	0 (0%)	1 (0.3%, *P*=0.328)	16 (5.5%)	3 (1.0%, *P*=0.057)	3 (1.0%, *P*=0.720)	3 (1.0%, *P*=0.466)	2 (0.7%, *P*=0.363)
Seg. 5	0 (0%)	0 (0%)	1 (0.3%, *P*=0.345)	3 (1.0%, *P*=0.057)	17 (5.8%)	9 (3.1%, *P*=0.000)	3 (1.0%, *P*=0.713)	6 (2.0%, *P*=0.001)
Seg. 6	0 (0%)	1 (0.3%, *P*=0.568)	1 (0.3%, *P*=1.00)	3 (1.0%, *P*=0.720)	9 (3.1%, *P*=0.000)	45 (15.4%)	27 (8.5%, *P*=0.000)	7 (2.4%, *P*=0.063)
Seg. 7	0 (0%)	0 (0%)	1 (0.3% *P*=1.00)	3 (1.0%, *P*=0.466)	3 (1.0%, *P*=0.713)	27 (8.5%, *P*=0.000)	40 (13.7%)	8 (2.7%, *P*=0.006)
Seg. 8	0 (0%)	0 (0%)	1 (0.3%, *P*=0.439)	2 (0.7%, *P*=0.363)	6 (2.0%, *P*=0.001)	7 (2.4%, *P*=0.063)	8 (2.7%, *P*=0.006)	23 (7.8%)

**Table 2:** Laboratory evaluations of patients who underwent surgery for the liver hydatid cyst.

Laboratory test		Value; median [Q1–Q3] or *n* (%)
White blood cell (10^3^/*µ*L)	Total	9.75 [7–12.7]
	Increased	139 (51.9%)
	Normal	125 (46.6%)
	Decreased	4 (1.5%)

Neutrophil (%)	Total	77.9 [54.7–87.1]
	Increased	38 (63.3%)
	Normal	18 (30%)
	Decreased	4 (6.7%)

Lymphocyte (%)	Total	12.2 [7.1–22.1]
	Increased	1 (1.7%)
	Normal	18 (30.5%)
	Decreased	40 (6738%)

Monocyte (%)	Total	5.9 [2.25–7.0]
	Increased	2 (11.8%)
	Normal	8 (47.1%)
	Decreased	7 (41.2%)

Eosinophil (%)	Total	10.0 [3.1–26.1]
	Increased	10 (62.5%)
	Normal	6 (37.5)
	Decreased	0 (0%)

Hemoglobin (g/dl)	Total	12.0 [11.0–13.4]
	Increased	9 (3.4%)
	Normal	100 (37.3%)
	Decreased	159 (59.3%)

Mean corpuscular volume (fL)	Total	82.9 [77.6–87.6]
	Increased	9 (4.7%)
	Normal	120 (63.2%)
	Decreased	61 (32.1%)

Platelet (10^3^/*µ*L)	Total	262.0 [199.5–350.0]
	Increased	34 (13.4%)
	Normal	188 (74.3%)
	Decreased	31 (12.3%)

Prothrombin time (sec)	Total	13.7 [13.0–14.8]
	Increased	31 (16.3%)
	Normal	139 (73.2%)
	Decreased	20 (10.5%)

Partial thromboplastin time (sec)	Total	33.0 [30.0–36.3]
	Increased	25 (13.9%)
	Normal	137 (76.1%)
	Decreased	18 (10.0%)

Blood urea nitrogen (mg/dl)	Total	11 [9.0–15.0]
	Increased	18 (6.9%)
	Normal	210 (81.1%)
	Decreased	31 (12.0%)

Creatinine (mg/dl)	Total	0.8 [0.7–1.0]
	Increased	43 (17.3%)
	Normal	196 (78.7%)
	Decreased	10 (4.0%)

Sodium (mEq/l)	Total	139 [136–141]
	Increased	4 (1.7%)
	Normal	179 (77.5%)
	Decreased	48 (20.8%)

Potassium (mEq/l)	Total	4.2 [3.9–4.5]
	Increased	15 (6.4%)
	Normal	201 (85.9%)
	Decreased	18 (7.7%)

Aspartate aminotransferase (IU/L)	Total	27.5 [19.75–41.0]
	Increased	53 (26.2%)
	Normal	143 (70.8%)
	Decreased	6 (3.0%)

Alanine transaminase (IU/L)	Total	24.0 [16.0–40.0]
	Increased	49 (24.5%)
	Normal	144 (72.0%)
	Decreased	7 (3.5%)

Alkaline phosphatase (U/L)	Total	249 [193–472]
	Increased	22 (10.7%)
	Normal	183 (88.8%)
	Decreased	1 (0.5%)

Total bilirubin (mg/dl)	Total	0.6 [0.4–0.8]
	Increased	22 (15.2%)
	Normal	121 (83.4%)
	Decreased	2 (1.4%)

Direct bilirubin (mg/dl)	Total	0.2 [0.1–0.3]
	Increased	21 (14.6%)
	Normal	121 (84.0%)
	Decreased	2 (1.4%)

Albumin (g/dl)	Total	4.2 [3.7–4.6]
	Increased	9 (9.4%)
	Normal	59 (61.5%)
	Decreased	28 (29.2%)

## Data Availability

The data used to support the findings of the study are available from the corresponding author upon request.
